# Students’ Conceptions of Animal Emotions and Cognitive Abilities in Karamoja (Uganda): Implications for Animal Welfare Education and One Health

**DOI:** 10.3390/ani16182812

**Published:** 2026-09-08

**Authors:** Eleonora Uber, Giacomo Riggio, Marta Becchetti, Maria Luisa Marenzoni, Laura Menchetti, Daniel Lolem, Mesfin Mekonnen Moliso, James Ocaa, Carlo Ruspantini, Pier Giorgio Lappo, Silvana Diverio

**Affiliations:** 1 Department of Veterinary Medicine, Perugia University, 06126 Perugia, Italy; eleonora.uber1@gmail.com (E.U.); giacomoriggio@gmail.com (G.R.); marialuisa.marenzoni@unipg.it (M.L.M.); mesfinareka@gmail.com (M.M.M.); 2 Cooperation and Development NGO (C&D), Moroto, Uganda; martabecchetti29@gmail.com (M.B.); daniellalolem@gmail.com (D.L.); ocaajames.ojj@gmail.com (J.O.); coopdev.lappo@hotmail.it (P.G.L.); 3 School of Biosciences and Veterinary Medicine, University of Camerino, 62024 Matelica, Italy; laura.menchetti@unicam.it; 4 Africa Mission-Cooperazione e Sviluppo, 29122 Piacenza, Italy; carlo.direzione@coopsviluppo.org

**Keywords:** animal emotions, animal cognition, students, pastoralism, Karamoja, Uganda, One Health, animal welfare, human–animal relationships, animal welfare education

## Abstract

Pastoralist communities in Karamoja maintain close, multifaceted relationships with animals from early childhood, yet little is known about how children conceptualise animal emotions and cognitive abilities. This pilot study explored these conceptions among Kasimeri Primary School students in Karamoja, Uganda, within the AICS All-In-One Project (AID 012590/04/1). A cross-sectional survey was conducted using a structured questionnaire administered to 326 students (mean age 13 ± 2 years). Data were analysed using descriptive statistics and generalised linear models (GLMs). Most children attributed a wide range of emotions to animals, with anger (79.6%) and happiness (78.2%) being the most widely recognised and disgust the least attributed (63.2%). Emotion ascription differed significantly by emotion type (*p* < 0.001) and species (*p* = 0.004), with significant interactions (*p* = 0.022). Species differences emerged for several emotions (all *p* ≤ 0.006), with ruminants and chickens receiving higher attribution than dogs, reflecting different species’ roles in pastoral life. Students from pastoralist households showed lower emotion scores than those with non-farmer fathers (*p* = 0.025). Cognitive ability attribution also differed significantly across species (all *p* < 0.001). These findings challenge Western-pet-centred frameworks, highlighting the need for culturally grounded, One Health-oriented educational approaches integrating local animal knowledge with formal welfare and health curricula.

## Simple Summary

In the Karamoja region of north-eastern Uganda, children grow up in close daily contact with livestock such as cattle, goats, and sheep, which are central to their families’ livelihoods and cultural identity. Despite this deep bond, little is known about how these children perceive and understand the inner lives of animals, whether they believe animals can feel emotions, think, or communicate. This study aimed to explore these perceptions by asking primary school students to answer questions about the emotions, abilities, and roles they attribute to different animal species. Results showed that the vast majority of students believe animals experience a wide range of emotions, including happiness, fear, anger, and sadness, and recognise their cognitive and communicative abilities. Students described animals’ emotional states mainly through behaviours observed daily, such as running away when scared or playing when happy. Interestingly, pupils from farming families tended to view animals in more practical terms rather than purely emotional ones. These findings are valuable because they reveal a rich, experience-based knowledge of animals that is largely absent from school curricula. Recognising and building on this knowledge could help develop more effective and culturally respectful education programs linking animal care, animal health, and human wellbeing.

## 1. Introduction

Pastoralist communities in Africa rely on a close and multifaceted relationship with animals. In regions such as Karamoja, north-eastern Uganda, livestock constitutes far more than an economic asset. It represents social capital, cultural identity, and a source of emotional attachment [1,2,3]. Children are deeply embedded in this pastoral system, assuming responsibilities for herding, milking, and caring for animals from an early age [4,5]. Through daily practices and playful imitation, they acquire skills and values that shape their transition into adulthood [6,7]. School attendance often alternates with livestock-related responsibilities, and educational pathways may therefore be irregular, reflecting the seasonal and socio-economic demands of pastoral life.

Although children’s perceptions of animals have been investigated in several cultural contexts [8,9,10,11], very little is known about how children growing up in pastoralist societies conceptualise animals’ emotions and cognitive abilities [12], where daily interactions with livestock constitute a fundamental component of social and cultural life. Studies in East Africa show that children learn about livestock not only through direct tasks but also through “herding games,” where they role-play human–animal interactions and reinforce cultural knowledge [6,7]. In Karamoja, flexible education initiatives such as the Alternative Basic Education for Karamoja (ABEK) program have demonstrated that integrating livestock knowledge into curricula enhances participation and bridges the gap between formal schooling and pastoral life [13,14]. These examples highlight how animals are central to both informal and formal learning processes. However, these approaches rarely investigate how children conceptualise animals’ inner states, such as emotions, intentions, or the capacity to feel.

This survey seeks to address this gap by exploring how students in Karamoja perceive animals and whether they attribute emotional and cognitive capacities to them.

By foregrounding students’ perspectives within the broader socio-ecological dynamics of Karamoja, this study contributes context-specific insights into the human–animal interface in pastoral communities. In a setting where children are involved in livestock husbandry from an early age, understanding how they perceive animals may help inform culturally grounded educational approaches. Children’s perceptions of animals are not merely a cultural curiosity. They may influence the way future adults interact with animals, recognise signs of pain, distress or disease, adopt husbandry practices, and engage with animal welfare and public health interventions. Understanding these perceptions is therefore relevant within a One Health framework, where human behaviour, animal welfare, livestock health and community wellbeing are closely interconnected. In fact, previous research has highlighted the importance of integrating One Health concepts into educational settings across Africa [15]. Incorporating these perspectives into educational programmes may support the design of locally relevant initiatives that promote animal welfare, livestock health, and community wellbeing in an integrated and culturally meaningful way. In this context, understanding children’s perceptions is particularly relevant because they represent the future livestock keepers, caregivers, and decision-makers within pastoral communities.

Accordingly, the aims of this study were: (1) to explore how children living in a pastoralist community attribute emotions and cognitive abilities to different domestic animal species; (2) to investigate whether these perceptions vary according to species and children’s socio-demographic characteristics; (3) to discuss the implications of these findings for culturally grounded animal welfare education within a One Health perspective.

## 2. Materials and Methods

### 2.1. Ethical Considerations

Students participated voluntarily during regular school activities. Prior to data collection, teachers and parents or legal guardians were informed according to the procedures routinely adopted by the local school. Considering the local cultural context, characterised by variable levels of literacy and by practices in which written consent is not routinely used, informed consent was obtained orally from parents or legal guardians following the procedures normally adopted by the school. Only students whose participation had been authorised were included in the survey. Students were also informed that participation was voluntary and that they could decline to answer any question without any consequence.

At the time of data collection, the activity was conducted as part of educational and awareness-raising initiatives within the ALL-IN-ONE project (see below), and was not originally designed as scientific research. Before statistical analysis, the dataset was pseudonymised by removing all personal identifiers. Subsequently, the secondary use of the anonymised data for scientific purposes received a favourable retrospective ethical opinion from the University of Perugia Bioethics Committee (Protocol No. 20/2026).

### 2.2. Study Area and Target Population

This research was conducted within the framework of the project “ALL IN ONE” “Integrated Actions in the Health, Sanitation and Livestock Sector to Respond to Epidemic-Prone Diseases with a One Health Approach” (AID 012590/04/1), funded by the Italian Agency for Development Cooperation (AICS), and led by the non-governmental organisation Africa Mission, Cooperation and Development (C&D), with the Department of Veterinary Medicine of the Perugia University (DVM-Unipg) as a project partner. The project aims to strengthen community resilience to epidemic-prone diseases in the Karamoja region through integrated interventions in human health, sanitation, and livestock management, recognising the interconnections between human, animal, and environmental health. The Karamoja sub-region is predominantly inhabited by agro-pastoralist communities in which livestock represents a fundamental source of livelihood, food security, cultural identity, and social value. Children are commonly involved in livestock-related activities from an early age, making this setting particularly suitable for investigating their perceptions of animal emotions and cognitive abilities.

Although livestock ownership varies among households, children in Karamoja are routinely exposed to a wide range of domestic animal species through daily village life and community activities, regardless of whether their own family owns those animals. Consequently, even children from non-pastoralist households are generally familiar with the behaviour and management of domestic animals, including non-farm animals, through frequent interactions within the community.

### 2.3. Data Collection

A descriptive, cross-sectional study design was employed at the Kasimeri Primary School in Moroto, Karamoja region (Uganda) between July and August 2024. The target group consisted of students aged approximately 10–14 years (Primary 5 class). However, some younger and older students were also present because age-grade heterogeneity is common in Karamoja, where school attendance may be interrupted or delayed by pastoral responsibilities and socio-economic factors.

**Figure 1:**
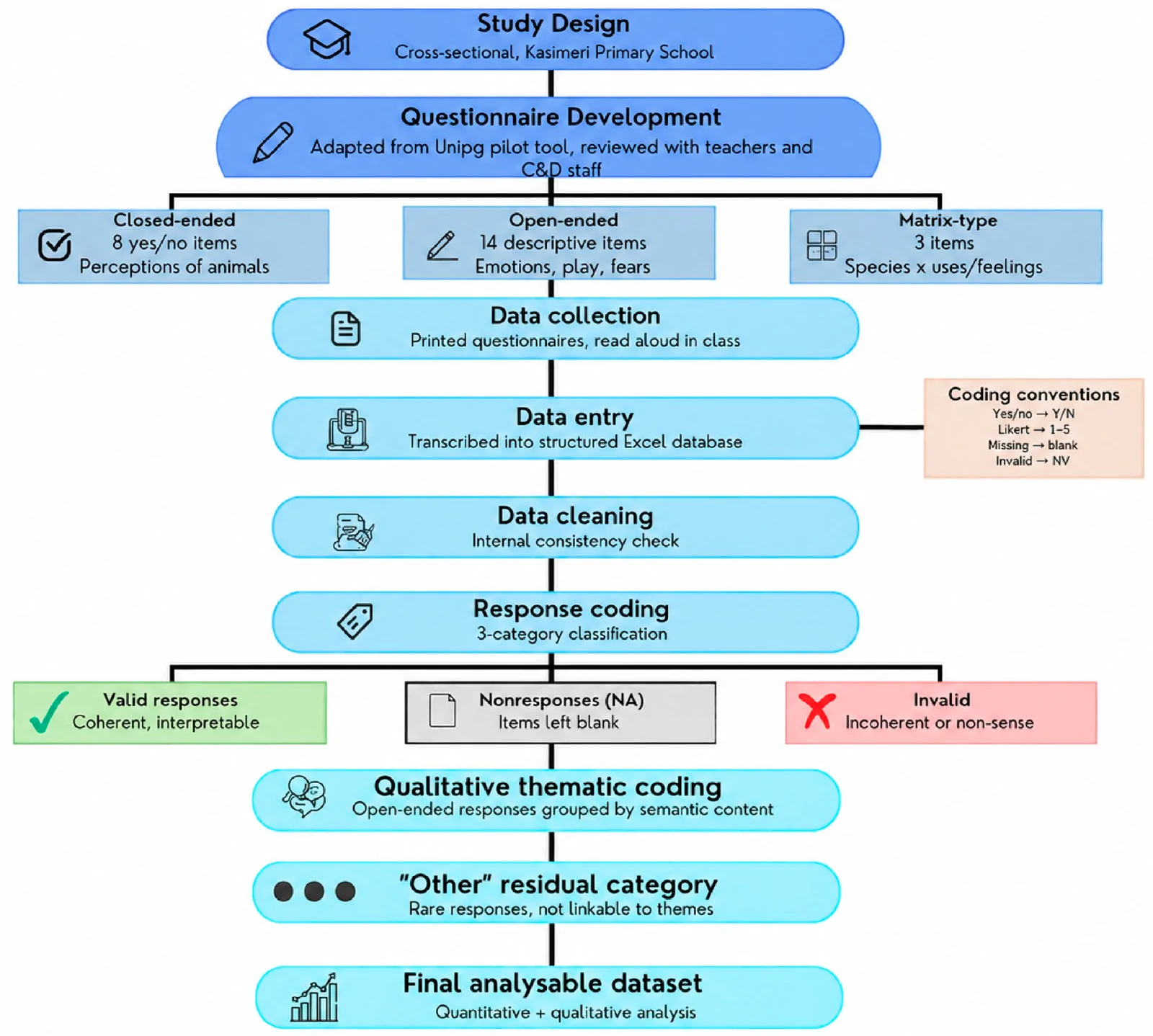
Flowchart describing the process from data collection to final cleaned dataset.

Data were collected using a structured questionnaire administered to students to explore their perceptions of animals’ emotions, cognitive and communicative abilities at a single point in time. The structure of the data collection and coding process is described in the following paragraphs and depicted in Figure 1.

### 2.4. The Questionnaire

The questionnaire consisted of demographic questions, closed yes/no items, matrix questions exploring attribution of emotions and cognitive abilities across animal species, Likert-scale items, and open-ended questions designed to explore children’s spontaneous representations of animal emotions, behaviour and human–animal interactions.

The questionnaire (Document S1) was adapted from a tool previously piloted by DVM-Unipg in other Ugandan schools [16] and further refined for the present study by researchers from DVM-Unipg, with the support of C&D staff and local school teachers who contributed their pedagogical expertise to ensure cultural relevance and comprehensibility. It was specifically designed for the objectives of the present study by combining questions adapted from previous educational and animal welfare surveys with additional items developed to investigate children’s perceptions of animal emotions, cognitive abilities, and human–animal interactions within the pastoralist context of Karamoja.

The questionnaires were administered collectively during class time by DVM-Unipg researchers, trained C&D staff, and local linguistic facilitators. The questionnaires were printed due to the absence of computers or other technological devices accessible to students. Considering the multilingual context, each question was read aloud and explained verbally in the local language by the facilitators (C&D staff), with the support of teachers, to ensure that all students fully understood the questions, across varying literacy levels, before completing the questionnaire independently.

Given the local context, characterised by variable and generally low literacy levels and by local practices in which written consent is not commonly used for this type of school activity, parents or guardians were informed about the study and their informed consent was obtained orally before student participation, following the procedures normally adopted by the local school institution. No parent or guardian declined to provide consent. Before data collection, permission from the gatekeeper to conduct the study was also obtained orally from the head of the school, in accordance with local institutional practices. Only students whose participation had been authorised were included. Participation was strictly voluntary, and students retained the right to decline participation or to leave any question unanswered. No sensitive personal data were collected or retained.

As already mentioned, this data collection was conducted within the framework of the ALL IN ONE Project, which involved collaboration with local partners and the prior engagement and agreement of the participating school institutions.

The questionnaire consisted of five sections, hereby briefly described.

The first section collected socio-demographic information, including age, sex, family composition, language spoken at home, daily activities, livestock ownership, and the perceived importance of animals for the family.

The second section explored children’s relationships with animals through closed- and open-ended questions investigating animal communication, emotional expression, attachment to animals, play behaviour, fear of dogs, and school education on animals. Questions on fear of dogs were included because dogs are among the domestic animals most frequently encountered by children in their daily lives, providing additional context for interpreting their perceptions of animals and human–animal relationships.

The third section included matrix-type items assessing the attribution of emotions to six domestic animal species (cattle, sheep/goats, chickens, donkeys, dogs, and cats).

The fourth section assessed children’s attribution of cognitive and social abilities to the same species through a second matrix of behavioural and cognitive traits.

The fifth section included scenario-based multiple-choice questions evaluating children’s knowledge of safe interactions with unfamiliar dogs.

The complete questionnaire is provided as Supplementary Document S1.

### 2.5. Data Coding and Analysis

Responses were coded into three categories:

1. Valid responses: coherent and interpretable answers aligned with the intended meaning of the question;
2. Non-responses (NA): items left unanswered;
3. Non-interpretable responses: incoherent, contradictory, or out-of-context answers.

This coding procedure allowed for clearer discrimination of data quality while retaining information on comprehension difficulties.

All questionnaires were transcribed into a structured Excel database, where closed-ended items (binary, matrix-type, and Likert-type) were coded numerically using predefined admissible values (respectively 1/0 and from 1 to 5). Missing answers were left as blank cells, while uninterpretable responses were flagged as NV (“non-valid”) to distinguish them from true non-responses. Open-ended responses were first transcribed verbatim and then inductively grouped into thematic categories based on shared semantic content (consultable in Table S1). A residual “Other” category was retained for low-frequency responses that did not fit any established theme. The data coding and cleaning procedure is displayed in Figure 1, and full details are provided in Document S2.

### 2.6. Statistical Analysis

Descriptive statistics were used to summarise the data as numbers and percentages, means and standard deviations (SD), and minimum and maximum values.

Differences in the uses and social roles attributed to animal species were analysed using generalised linear models (GLMs) with a binomial distribution and logit link function. Because each participant evaluated multiple animal species, repeated responses were accounted for using generalised estimating equations (GEEs) with an exchangeable working correlation structure. Animal species was included as the explanatory factor, and pairwise comparisons among estimated marginal means were performed where appropriate. Results are presented as numbers of affirmative responses (n), estimated marginal probabilities (%), and their corresponding 95% Wald confidence intervals (95% CIs), together with Wald χ^2^ test *p*-values. The same modelling approach was used to analyse differences in the attribution of emotions, with emotion type, animal species, and their interaction included as explanatory factors. Results are presented as estimated marginal means together with the corresponding Wald χ^2^ test *p*-values. Factors associated with fear of dogs were analysed using GLM with a binomial distribution and logit link function. Results are presented as regression coefficients (β ± standard error [SE]) together with the corresponding Wald χ^2^ test *p*-values.

For each participant, an *Animal Emotion Score* was calculated as the percentage of affirmative responses across the 72 emotion-related items included in Question 15 (12 emotional states evaluated for each of the six animal species, i.e., happy, love, angry, scared, surprised, disgusted, lonely, sad, hateful, jealous, satisfied, and pain perception). Similarly, an *Animal Competence Score* was calculated as the percentage of affirmative responses across the 72 competence-related items included in Question 16 (12 cognitive and functional attributes evaluated for each of the six animal species, i.e., defend the herd, defend themselves, feel love for their farmer, find a source of water, help each other, mothers recognise their offspring, recognise animals from the same herd, recognise danger, recognise their farmer, return alone to the village, take care of other animals, and take care of themselves). Both scores ranged from 0 to 100, where 0 indicated no affirmative responses and 100 indicated affirmative responses to all items; higher scores indicated a greater tendency to attribute emotions or cognitive abilities to animals, respectively. Associations between the two scores and students’ age, sex, paternal farming occupation, and perceived importance of animals to the family were evaluated using GLMs with a normal distribution and identity link function. Results are presented as estimated marginal means with their corresponding 95% Wald confidence intervals (95% CIs) and Wald χ^2^ test *p*-values.

Finally, for each questionnaire item investigating students’ intended behaviours towards dogs, pairwise associations among the dichotomous response options were evaluated using Pearson’s chi-square tests. For each pair of responses, the strength of the association was quantified by calculating the odds ratio (OR) and its 95% confidence interval (95% CI). To account for multiple pairwise comparisons within each questionnaire item, *p*-values were adjusted using the Benjamini–Hochberg false discovery rate (FDR) procedure. Associations with FDR-adjusted *p*-values < 0.05 were considered statistically significant.

All analyses were performed using SPSS software version 25 (SPSS Inc., Chicago, IL, USA), and *p*-values < 0.05 were considered statistically significant.

## 3. Results

**Figure 2:**
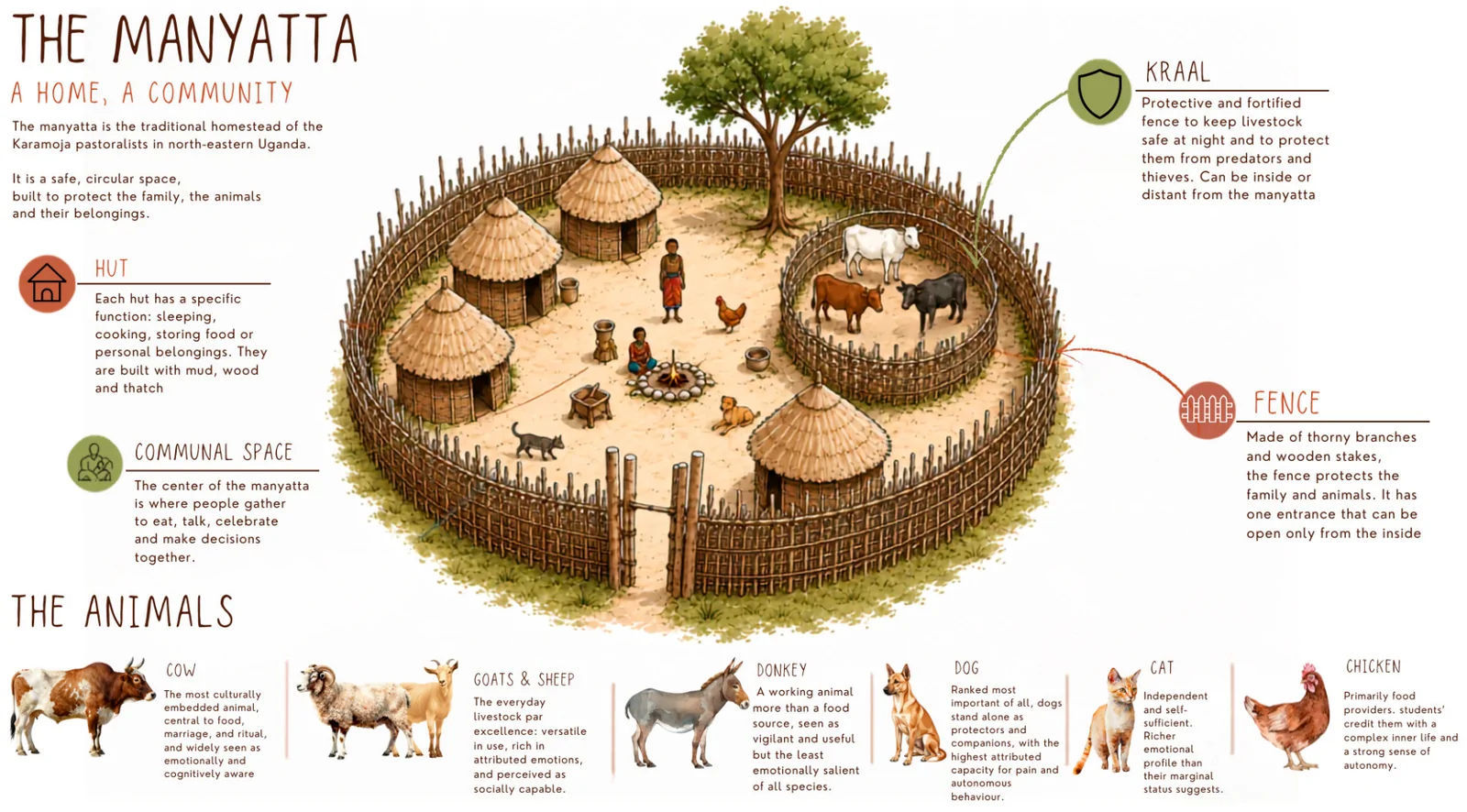
Schematic representation of a traditional Karamoja manyatta illustrating the spatial organisation of the socio-cultural homestead in which children interact daily with the main domestic animal species considered in the questionnaire (cattle, sheep/goats, chickens, dogs, cats, and donkeys), highlighting the embodied role and importance of each species.

The following section presents the results of the survey, organised according to the main thematic areas investigated: the descriptive characteristics of the participating students, their attitudes and beliefs toward animals. The roles that students attribute to different species are schematically summarised in Figure 2, which depicts the manyatta, the traditional homestead of the Karamoja pastoralist communities. This living space shared by families and the animals is central to this study, as shown in Figure 2, which provides a visual reference for understanding this cultural context.

### 3.1. Descriptive Characteristics of the Students

A total of 326 students were included in the study, aged between 8 and 21 years (mean ± SD = 13 ± 2 years). Of the 326 questionnaires, sex information was valid for 279 students, of whom 130 (46.6%) were female, and 149 (53.4%) were male. Sex information was invalid in 17 questionnaires (5.2%) and missing in 30 (9.2%).

Regarding family language, the largest proportion of students reported speaking Karamojong at home (114; 35.1%), followed by Ateso (100; 30.8%) and English (64; 19.7%). Other languages were less frequently represented (Table S2).

Most students lived with one or both parents (176; 70.1%), while a substantial proportion resided with other family members (62; 24.7%), indicating that nearly one in four children were cared for within the extended family. Regarding perceived family closeness, students most frequently identified their mother as the family member to whom they felt closest (156; 50.5%), followed by their father (75; 24.3%). Other family members were mentioned considerably less often (Table S2).

### 3.2. Students’ Attitudes Toward Animals

The majority of students reported that their parents were farmers (144; 50.7%). Regarding livestock ownership, most families owned cattle (110; 41.8%), followed by small ruminants (76; 28.9%), while only a minority owned cats (3; 1.1%) or dogs (13; 4.9%). Additionally, 8.7% of families owned more than one livestock species (Table S2).

Animals were considered highly important for the family by most of the students, with 70.1% (190 respondents) assigning the maximum score (5). The main reasons reported were food provision (125; 51.2%) and income generation (56; 23.0%), while 11.5% (28) mentioned other reasons and 11.1% (27) provided invalid or unclear responses (Table S2).

A large proportion of students reported studying animals at school: of the valid answers, 228 (79.2%) answered yes, while 60 (20.8%) stated that they do not. Among those who reported studying animals, the most frequently mentioned topic was taxonomy (82 students; 36.0%), followed by body parts, biology or physiology (47; 20.6%) and care, diseases, husbandry and animal products (42; 18.4%). Smaller proportions referred to behaviour, emotions and communication (12; 5.3%) or to general or unspecified response (10; 4.4%). A notable proportion of responses were classified as invalid or unclear (35; 15.4%).

Overall, most students (218; 78.1%) believed that animals can communicate with one another, whereas approximately one in five (61; 21.9%) did not share this view (Table S3). Among those who believed that animals can communicate, the most frequently mentioned modality was vocalisations (150; 62.2%), followed by body language or physical movements (29; 12.0%) (Table S3). Regarding students’ interpretations of animal emotional responses, anger was most commonly described as aggression or attack (84; 27.7%), whereas fear was primarily associated with escape or avoidance behaviours (136; 48.9%). Happiness was predominantly expressed through movement or play (143; 47.2%), while sadness was mainly characterised by withdrawal, isolation, or inactivity (73; 24.7%). Surprise was often interpreted as positive surprise, play, or increased movement (92; 35.9%). Responses regarding disgust were more heterogeneous, with anger/irritation (42; 18.9%) and miscellaneous reactions (42; 18.9%) being the most frequently reported descriptions (Table S3).

Most respondents reported a positive emotional orientation toward animals: 97.7% stated they love animals, while only 2.3% said they do not. Similarly, the majority (215; 82.1%) believed that animals can love humans, although 17.9% did not share this view. Among those who believed that animals can show love, dogs were the most frequently mentioned species (197; 65.0%), followed by ruminants (42; 13.9%) and cats (26; 8.6%) (Table S4).

As regards animal cognitive abilities, most students (81.7%) believed that animals are capable of thinking, while 18.3% did not share this view (Table S4). When asked about their favourite animal, cattle and small ruminants collectively accounted for the largest proportion of responses (47.2%), followed by dogs (31.5%) and cats (7.5%). Most participants reported playing with animals (232; 77.6%), primarily through active, movement-based play (125; 55.8%), followed by feeding-mediated play (34; 15.2%). A similar pattern emerged specifically for dogs; the answers to the question “*Do you play with dogs?*” registered 64.3% of students reporting that they play with them, again predominantly through active play (119; 58.6%) (Q: *If yes, how do you play with dogs?*). However, fear of dogs was common, with 65.8% of students reporting being afraid of them. Among these students, fear was mainly attributed to aggression (120; 60.6%). Notably, 17.2% provided positive statements, indicating that they did not fear dogs rather than describing a reason for fear or not fear (Table S4). Fear of dogs tended to decrease with age (β = −0.128 ± 0.074; *p* = 0.080) and was not associated with sex or whether the parents were farmers. However, fear appeared to influence interactions with animals, as it was strongly associated with whether participants reported playing with animals in general (β = −1.220 ± 0.302, *p* < 0.001) and with dogs specifically (β = −1.004 ± 0.292, *p* < 0.001).

### 3.3. Use of Different Animal Species

Clear differences emerged among animal species regarding the uses and roles attributed to them by students (Table 1). Sheep and goats were most frequently associated with the traditional practice of blood consumption (*p* < 0.001). Consumption-related uses varied significantly among species. Chickens were most frequently associated with the “*consumption of milk or eggs*”, followed by cattle and sheep/goats, whereas donkeys were rarely linked to this purpose (*p* < 0.001). Similarly, sheep/goats, chickens, and cattle were the species most commonly identified as sources of meat, while donkeys were least frequently mentioned in this context (*p* < 0.001).

Animals were widely perceived as playing important roles in cultural and social practices. Sheep/goats, cattle, and chickens were frequently associated with initiation ceremonies and marriage-related traditions, whereas donkeys were consistently less linked to these functions (*p* < 0.001).

Significant interspecies differences emerged regarding the use of animal-derived materials for clothing, bedding, and housing. Sheep/goats and cattle were most commonly cited for these purposes, while chickens were frequently mentioned only in relation to clothing. In contrast, chickens and donkeys were less often associated with housing-related uses (*p* < 0.001).

Village protection was predominantly attributed to dogs (70.4%), which were associated with this role significantly more frequently than any other species (*p* < 0.001). In contrast, sheep/goats, cattle, chickens, and donkeys were only rarely linked to protective functions.

Finally, ownership patterns differed significantly across species (*p* < 0.001). Chickens showed the highest proportion of students reporting not owning the species, whereas donkeys showed the lowest.

Overall, the results showed clear differences in the perceived roles of the different animal species, with each species being consistently associated with specific economic, cultural, or protective functions.

### 3.4. Effect of Type of Emotion and Species

Responses concerning emotions attributed to different animal species varied significantly according to emotion type (*p* < 0.001) and species (*p* = 0.004), with a significant emotion type × species interaction (*p* = 0.022; Table 2). Overall, irrespective of species, *anger* and *happiness* were the emotions most frequently attributed to animals (both >78% of respondents), whereas *disgust* was the least frequently attributed (<65%; *p* < 0.05).

Species differences emerged only for happiness, love, and fear (all *p* < 0.01). Happiness was more frequently attributed to sheep/goats and chickens than to the other species, with percentages exceeding 80%. Similarly, the capacity to feel love towards another animal was more often attributed to sheep/goats, cattle, and chickens than to cats and dogs, for which attribution rates were below 70%. Fear was also more frequently attributed to sheep/goats, cattle, and chickens than to dogs. Finally, multiple comparisons showed that jealousy towards another animal was more commonly attributed to chickens than to sheep/goats and cattle (Table 2).

**Figure 3:**
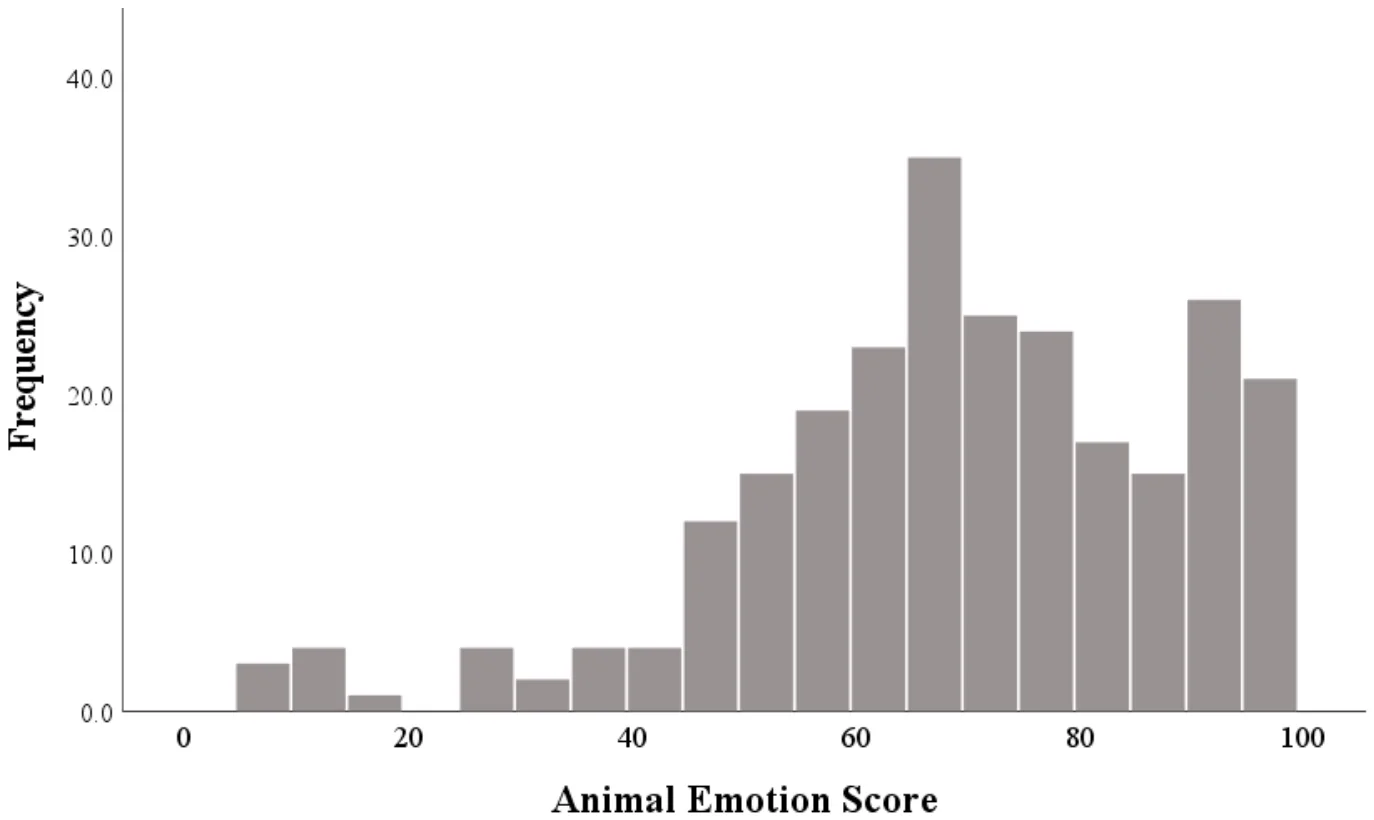
Distribution of the Animal Emotion Score among participating students. The Animal Emotion Score was calculated as the percentage of affirmative responses across all emotion-related questions and animal species. The histogram shows the frequency distribution of scores ranging from 0 to 100.

The distribution of the *Animal Emotion Score*, reflecting the overall tendency of students to attribute emotions to animals, is shown in Figure 3. Scores were generally high, with a median of 72.0 (25th percentile: 60.0; 75th percentile: 89.0). Most values were concentrated in the upper range of the scale, indicating a general tendency among students to attribute a broad range of emotions to animals.

The *Animal Emotion Score* was not associated with age. A trend towards a sex difference was observed, with females tending to have higher Animal Emotion Scores than males (73.8, 95% CI 70.2–77.3 vs. 69.4, 95% CI 66.1–72.7; *p* = 0.080). Students whose fathers were not farmers had slightly higher Animal Emotion Scores than those whose fathers were farmers (74.8, 95% CI 71.5–78.2 vs. 69.4, 95% CI 66.2–72.7; *p* = 0.025). In contrast, scores were not associated with the importance that students attributed to animals.

Overall, students attributed a broad range of emotions to all animal species, although the frequency of attribution varied according to both the type of emotion and the species considered.

### 3.5. Students’ Beliefs Regarding the Cognitive and Functional Abilities of Animals

Students generally attributed a wide range of cognitive and functional abilities to animals, with several abilities being consistently recognised across species (Table 3). Overall, more than 70% of respondents believed that animals can feel love for the farmer, that they can find a source of water, that mothers recognise their offspring, and that they can take care of themselves. The remaining abilities were nonetheless acknowledged by 60–70% of respondents.

Significant interspecies differences emerged for selected abilities. The capacity to find a source of water was most frequently attributed to sheep/goats, cattle, dogs, and donkeys than to cats and chickens (*p* < 0.001). Cats and dogs were most frequently perceived as capable of recognising danger compared with the other species (*p* < 0.001). Differences were also observed for the ability to return alone to the village, with dogs showing the highest percentages, whereas cattle and donkeys were least frequently associated with this ability (*p* < 0.001).

**Figure 4:**
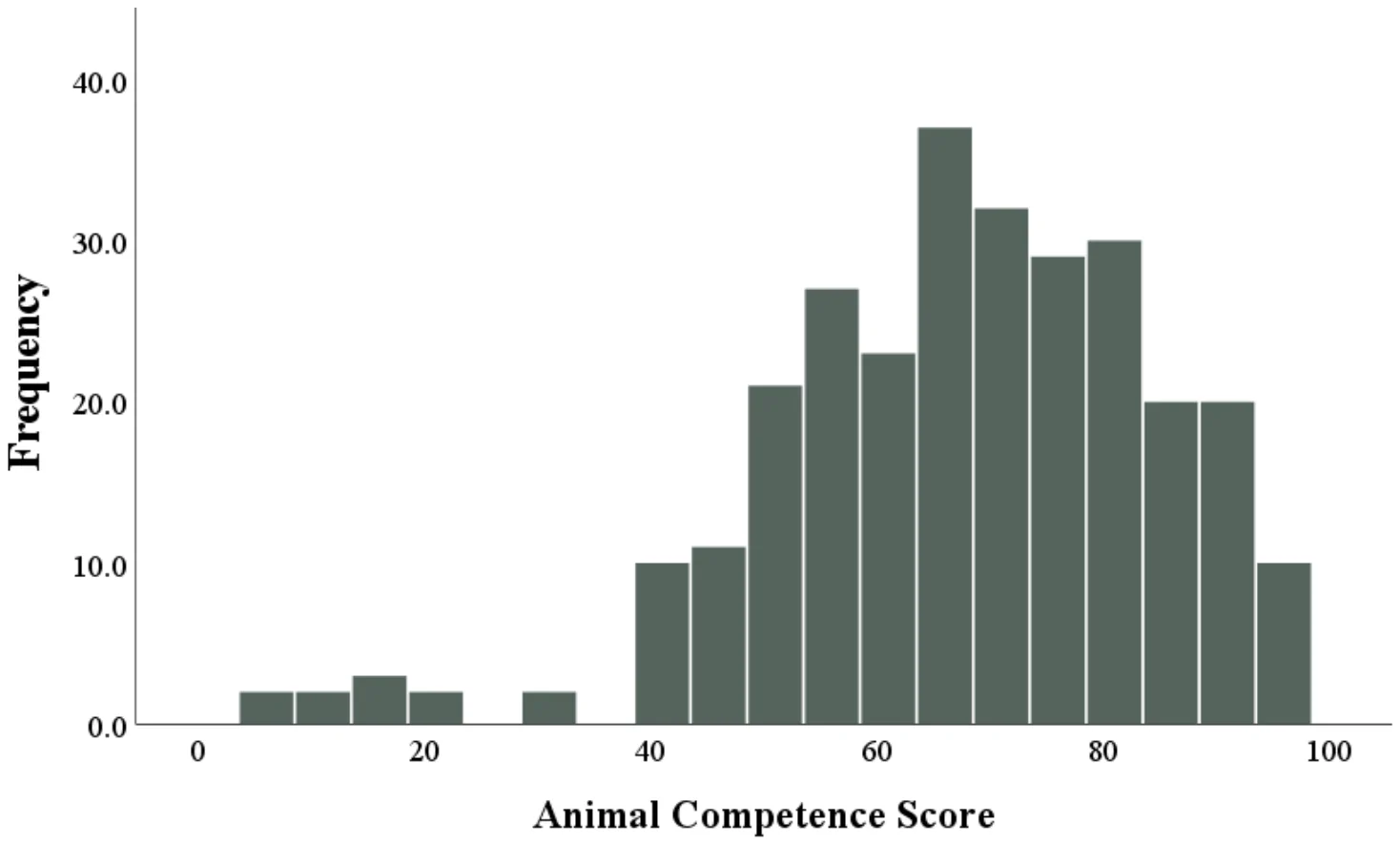
Distribution of the *Animal Competence Score* among participating students. The *Animal Competence Score* was calculated as the percentage of affirmative responses across all cognition- and ability-related questions and animal species. The histogram shows the frequency distribution of scores ranging from 0 to 100.

The distribution of the *Animal Competence Score*, representing the proportion of affirmative responses across all cognition- and ability-related questions and animal species, is shown in Figure 4. Scores were generally high, with a median of 71.0 (25th percentile: 58.0; 75th percentile: 83.0), indicating that students attributed a broad range of competences to animals in most of the items considered.

Among the demographic variables analysed, only a trend related to the father’s occupation was observed. Students whose fathers were not farmers tended to have higher *Animal Competence Scores* than those whose fathers were farmers (72.4, 95% CI 69.1–75.7 vs. 67.8, 95% CI 64.6–71.1; *p* = 0.053).

Overall, students attributed numerous cognitive abilities to all animal species, with relatively limited differences among species for most of the abilities investigated.

### 3.6. Students’ Approach to a Dog

Students reported a wide range of behaviours when asked how they would approach an unfamiliar dog (Table S5). Participants could select multiple responses, including potentially contradictory behaviours, such as approaching and avoiding the dog. The most frequently reported behaviour was asking the owner for permission before touching the dog (74.0%), followed by touching the dog’s head (63.7%), calling the dog gently (58.8%), letting the dog sniff one’s hand before touching it (49.5%), not approaching the dog (45.3%), and chasing the dog away (42.6%) (Table S5). Pairwise co-occurrence analyses identified eight significant associations after Benjamini–Hochberg false discovery rate correction (Table 4). Letting the dog sniff one’s hand before touching it was associated with greater odds of also asking the owner’s permission, not approaching the dog, and chasing the dog away. Students who reported asking the owner’s permission were more likely to also report that they would not approach the dog. Similarly, chasing the dog away was associated with not approaching it. Calling the dog gently was associated with touching the dog’s head, asking the owner’s permission, and not approaching the dog.

Answers to question “*What would you do if faced with a growling dog?*” showed a similar pattern. The most frequently endorsed strategy was looking for help (74.7%), followed by running away (64.9%), staying still without looking the dog in the eyes (54.4%), trying to calm the dog by talking gently (46.8%), and trying to fight the dog off (29.1%) (Table S6). Co-occurrence analyses (Table 5) identified only two significant associations after adjustment for multiple comparisons. Students who reported that they would run away were substantially more likely to also report that they would look for help (FDR-adjusted *p* = 0.003) and try to fight the dog off (FDR-adjusted *p* = 0.003). No other pairwise associations remained significant after false discovery rate correction.

Overall, running away was significantly associated with both seeking help and attempting to fight the dog, whereas no other response strategies showed significant associations.

## 4. Discussion

This survey provides an initial, descriptive exploration of how children and youth living in the pastoral context of the Karamoja region perceive animals’ emotions and cognitive and communicative abilities. Rather than measuring empathy as a psychological trait, the present findings capture how students articulate knowledge about animals through emotionally and functionally framed responses. This distinction is critical, as much of the existing literature on students’ relationships with animals is grounded in Western, urban, and pet-centred frameworks that prioritise affective attachment or moral concern as primary indicators of human–animal relationships [16,17]. In contrast, the Karamoja context requires a different interpretative lens, one that recognises students as embedded actors within a pastoral socio-ecological system, where animals are integral to subsistence, safety, and everyday life and where animal-related knowledge is primarily acquired through everyday experience.

### 4.1. Emotional Attribution as Behaviour-Based and Situated Knowledge

Students consistently attributed emotional states to animals, with high overall percentages for commonly recognised emotions such as anger, happiness, sadness, and fear. In contrast, more complex emotions requiring a finer interpretation of behavioural cues, such as disgust, were attributed less frequently. Emotional attributions varied primarily according to emotion type rather than animal species. When asked how animals express emotions, participants predominantly referred to observable behaviours, including aggression for anger, escape or avoidance for fear, movement or play for happiness, and withdrawal for sadness. Many of these descriptions are broadly consistent with commonly recognised behavioural expressions of the corresponding emotions [18], suggesting that children’s interpretations are grounded in recognisable behavioural patterns. This finding is consistent with developmental and comparative research showing that children often rely on perceivable cues, such as movement, vocalisation, or avoidance, to infer emotional states in both humans and animals [19].

In pastoral settings such as Karamoja, where children interact daily with livestock and working animals [13,14], the ability to interpret behavioural signals is not only a cognitive process but also a practical necessity. Recognising signs of fear, aggression, or distress may have direct implications for personal safety, animal management, and effective human–animal interactions. Notably, this form of emotional and behavioural knowledge appears to be largely acquired outside formal education. In the present sample, only 5.3% of school-based animal-related teaching addressed behaviour, emotions, or communication, whereas curricular content focused predominantly on taxonomy and biological or productive aspects. Although these data are based on the respondents’ spontaneous recall, the gap they suggest between formal education and practical knowledge is consistent with findings in pastoral communities elsewhere, such as in south-western Uganda and Kenya, where livestock keepers possess sophisticated embodied knowledge of cattle acquired through daily practice rather than formal instruction [20,21]. Similar patterns have been documented among Maasai children, who acquire animal-related knowledge through daily herding activities and play [7], suggesting that experiential learning is a common pathway through which such knowledge develops in pastoral societies.

The present findings may be interpreted through the concept of “situated knowledge” proposed by Haraway [22], whereby knowledge is understood as partial, positioned, and grounded in specific relationships and everyday practices. Within this perspective, children’s understanding of animal emotions appears to be shaped by their daily interactions with livestock and working animals rather than by abstract or universal concepts of animal behaviour. Such knowledge should not be regarded as inferior to scientific knowledge; rather, it represents a different way of understanding animals that serves distinct practical functions within a specific socio-cultural context. Recognising this perspective encourages researchers to avoid interpreting pastoral children’s understanding of animals exclusively through urban, pet-centred frameworks. Similar perspectives have also been described in ethnoveterinary and anthropological studies of pastoral communities, although primarily from epidemiological and animal health perspectives [20,21,23,24]. Our findings are consistent with the literature and further suggest that children’s everyday interactions with livestock may contribute to shaping their perceptions of animal emotions and cognition, as well as the ways in which this knowledge is expressed.

At first glance, the lower *Animal Emotion Scores* observed among students from pastoralist households may appear to challenge the interpretation that understanding animal emotions is primarily grounded in direct interaction. However, rather than contradicting this assumption, these findings may suggest that contact does not uniformly increase the attribution of emotions; instead, it may influence how animals are perceived within specific socio-ecological contexts. As shown by our results, livestock often function as key economic assets, representing a form of “living capital” within pastoral livelihoods [25]. Within this framework, children may develop a more functional and pragmatic understanding of animals, focusing on behaviour, productivity, and management rather than on the explicit attribution of internal emotional states. This interpretation aligns with evidence suggesting that moral reasoning in resource-dependent settings is often shaped by utilitarian and consequence-based considerations, particularly in communities experiencing increasing market integration, a dynamic documented across several pastoral systems [26]. In such environments, animals are not only relational beings but also essential components of livelihood strategies. These factors may encourage forms of reasoning that prioritise practical outcomes alongside, or sometimes over, affective interpretations. Consequently, lower levels of explicit emotion attribution should not necessarily be interpreted as reduced sensitivity, but rather may reflect an adaptive mode of human–animal relationship.

It is worth noting that the difference between children of farmers and non-farmers was statistically significant only for emotion attribution and not for cognitive-ability attribution, and that even for emotion attribution the difference was modest, with both groups attributing emotions at consistently high rates. The following interpretation should therefore be read as an attempt to account for a relatively small, emotion-specific difference rather than a broad divide. The divergence in how students perceive animals may be traced to the nature of their interaction with the natural world. While direct involvement in livestock management is likely to foster a pragmatic, experience-based understanding, those with less direct involvement may rely more heavily on socially transmitted and generalised representations of animals, which often emphasise emotional capacities and anthropomorphic interpretations [27,28]. This distinction is particularly relevant when considering the methodology used in the present survey. The assessment tool measured explicit and verbalised attributions of emotions, which may be more closely aligned with representational forms of knowledge than with the tacit understanding acquired through everyday experience and practical engagement with animals. Taken together, these findings may suggest the coexistence of multiple ways of knowing animals: one that is behaviour-based, situated, and practice-oriented, and another that is more abstract, generalised, and verbally articulated. Rather than being mutually exclusive, these perspectives may be shaped by different combinations of experience, socialisation, and cultural context, each providing valuable but distinct insights into human–animal relationships. Although this interpretation is consistent with the present findings and with evidence from comparable pastoral contexts, it should be regarded as a theoretical framework rather than as a direct empirical conclusion. The study did not directly investigate children’s daily interactions with animals, nor did it examine in depth the processes through which animal-related knowledge is acquired, shared, and transmitted within the community. Consequently, the proposed interpretation is based on the convergence of empirical findings from the present survey, evidence from comparable pastoralist contexts, and the ecological realities of livestock-based livelihoods, suggesting that children’s responses reflect a culturally embedded form of situated behavioural knowledge.

Future research employing ethnographic, observational, and participatory approaches would be valuable for exploring these learning processes in greater depth and for further assessing how children in Karamoja develop their understanding of animals through everyday experience. Such approaches would also help distinguish more clearly between behavioural knowledge acquired through practice and children’s explicit representations of animal emotions and cognition.

### 4.2. Beyond Speciesism: A Relational Understanding of Animals

While emotion type was the strongest predictor of emotion attribution, significant species differences emerged for specific emotions and cognitive abilities. Importantly, these patterns did not mirror those commonly reported in Western societies, where companion animals typically receive greater emotional consideration than livestock [11,17,29]. In contrast, ruminants occupied a central position in the perceptions of the students surveyed. They were the animals most frequently identified as favourites, were more often attributed the capacity to feel love towards other animals, and received high ratings across a range of emotional capacities. This pattern may reflect the economic and cultural importance of ruminants within Karamoja pastoral livelihoods, where children interact with them daily through herding and husbandry activities from an early age [4,5]. Their prominent role within pastoral households is reflected by their being the most commonly owned animals. Taken together, these findings suggest that emotional attribution may depend less on species category (e.g., “companion” in a westernised conception versus farm animal) than on the nature, frequency, and cultural significance of human–animal interactions within a given socio-ecological context, consistent with observations from other pastoral societies [7,20,21].

Dogs presented a more complex pattern. While strongly associated with village protection and the capacity to love humans, they were often feared by most students, primarily due to concerns about aggression. This ambivalence likely reflects two factors: (1) relatively low household dog ownership, meaning students frequently encounter free-roaming rather than owned dogs, and (2) legitimate safety concerns in a region where rabies and dog-mediated injuries pose real risks [30,31]. These findings contrast with the “speciesist gradient” described in many Western populations [11,32], where companion animals consistently receive greater moral and emotional consideration than livestock. Rather than revealing a clear hierarchical ranking of species, our findings may instead suggest a pattern of functional differentiation. Ruminants received high emotional attribution across most dimensions, dogs were recognised for specific capacities such as protection and danger recognition, and different species were associated with distinct emotional and cognitive abilities. These findings may indicate that animals were evaluated according to their perceived roles and relationships with humans, rather than along a single continuum of moral or emotional worth.

This pattern may be interpreted as consistent with anthropological accounts of human–animal relational ontologies, in which species are not organised within a hierarchical framework but occupy distinct positions within networks of multispecies relationships [33]. Within this interpretative framework, children’s attribution of animal emotions may reflect differences in lived interactions and social relationships with animals rather than speciesist evaluations. This interpretation is further supported by the pattern observed for cognitive abilities, where children attributed specific competencies to specific species depending on the task considered (Table 3), rather than around a general ranking of intelligence across species. Notably, this species- and task-specific pattern emerged despite the clear differentiation in the practical, economic, and cultural roles assigned to animal species (Table 1). In this study, we did not ask children to rank animals by overall worth or ability, and we therefore make no claim about a hierarchy of value. What our data show is that recognised competence was distributed across species according to task rather than concentrated in any one ‘most capable’ animal. Taken together, these findings may therefore suggest a relational understanding of animals that is not grounded in (Western) pet-centred models of emotional proximity, but rather in animals’ community roles, behaviours, and everyday interactions within the pastoral socio-economic system, as described in other anthropological accounts of human–animal relations in pastoral and non-Western contexts [33,34,35,36].

Much of the existing literature on children’s relationships with animals employs empathy as a central construct, typically measured through affective scales developed and validated in Western, urban, pet-keeping societies [37,38]. However, the present survey did not measure empathy as a psychological trait. Rather, it explored how children describe and interpret animals in emotional and behavioural terms. This distinction is important. Students’ recognition that animals can experience emotions such as fear, anger, and happiness, together with their ability to identify behavioural expressions associated with these emotions, may be interpreted as reflecting a form of emotional understanding regarding animals. However, this should not be equated directly with empathy as commonly measured by standardised instruments, which often emphasise affective identification, emotional concern, or sentimental attachment.

In pastoral systems, care for animals may be expressed through competent and responsible practice—ensuring access to water, recognising signs of distress, and responding appropriately to behavioural cues—rather than through overt expressions of affection [39]. Thus, although the recognition of emotions in animals may provide a foundation for empathic awareness [40], it does not necessarily imply the adoption of an empathic orientation towards animals. The relationship between emotion recognition, empathy, and caregiving practices therefore warrants further investigation in pastoral contexts. Future studies combining behavioural observations with validated measures of empathy and human–animal relationships would help clarify how these dimensions interact across different cultural and socio-ecological settings.

### 4.3. Implications for Animal Welfare Within a One Health-One Welfare Perspective

The high levels of emotional attribution observed among participants, together with their ability to recognise behavioural indicators of emotional states, suggest a promising foundation for welfare-oriented educational interventions. Rather than basing these initiatives on externally derived, pet-centred notions of animal welfare, such strategies in pastoral settings may be more effective when built upon locally grounded ways of perceiving and relating to animals. This perspective is consistent with recent calls to rethink animal welfare through culturally sensitive and context-specific lenses, recognising that human–animal relationships, ethical priorities, and welfare practices are shaped by social, historical, and political contexts rather than universal standards [41]. Children’s existing knowledge therefore represents a valuable starting point for developing culturally appropriate educational programmes that strengthen animal welfare while remaining socially relevant.

This approach resonates with the One Health-One Welfare framework, which emphasises the interdependence of animal welfare, human well-being, and environmental health, and advocates integrated, multi-sectoral responses that transcend disciplinary boundaries [42]. Evidence from diverse contexts shows that systemic welfare deficits (e.g., inadequate veterinary coverage, limited disease surveillance, and risky human–animal practices) can contribute directly to broader public health challenges, including the persistence of zoonotic diseases [43,44,45]. In sub-Saharan Africa, where livestock play a central role in livelihoods, but animal welfare is often structurally constrained by limited resources and services, welfare outcomes are shaped by a complex interplay between productivity needs, caregiving practices, and the values attributed to animals [46,47]. Within this context, recognising students’ existing knowledge of animal behaviour does not imply that welfare standards are (already) adequate. Rather, it highlights an opportunity to develop culturally sensitive interventions that build upon local knowledge systems and strengthen animal care practices without undermining the socio-economic realities of pastoral communities. Educational initiatives that integrate students’ existing behavioural knowledge with animal welfare, disease prevention, and safe human–animal interactions may reinforce children’s practical understanding while promoting healthier relationships between people and animals. By building on existing local knowledge, educational programmes have the potential to strengthen both animal welfare and human health while remaining culturally appropriate and socially sustainable.

More broadly, understanding children’s perceptions of animal emotions and cognitive abilities is particularly important as they will likely shape future human–animal interactions, livestock management, and attitudes towards animal welfare. Integrating scientific evidence with children’s behavioural knowledge may support culturally appropriate One Health-One Welfare interventions that strengthen animal welfare while benefiting both animals and people.

### 4.4. Limitations

Some limitations constrain the interpretation and generalizability of our findings. Among these, the cross-sectional design we employed in this survey captured students’ beliefs at a single point in time, preventing conclusions about developmental changes or the effects of accumulated experience. Future longitudinal studies would help clarify how practical experience with animals shapes animal-related knowledge and perceptions over time.

A further limitation concerns the sampling strategy. Participants were recruited through a school-based survey, which may have overrepresented children from families prioritising formal education and underrepresented those engaged full-time in herding or belonging to highly mobile pastoralist households. In addition, data were collected in a single primary school. Although students originated from different villages and settlements, and schools in Moroto often serve a wide catchment area due to the limited educational infrastructure in the region, the sample cannot be considered fully representative of the diversity of experiences across Karamoja’s nine districts. Furthermore, information on urban versus rural residence was not systematically collected, preventing a more detailed analysis of how living environments may influence children’s perceptions of animals. Future studies involving multiple schools across different districts would help capture the heterogeneity of pastoral and semi-urban contexts within the region.

Another limitation concerns questionnaire comprehension and response format. A relatively high proportion of invalid or incomplete responses was observed for some emotion attribution questions (14–27% depending on the item). These missing responses were concentrated in matrix-format questions, which required participants to evaluate multiple emotions across different animal species within the same table. Although the emotional concepts appeared to be generally understood, as suggested by the consistency of valid responses, the matrix structure may have represented an unfamiliar format for some students. Consequently, invalid responses likely reflect difficulties with the questionnaire layout rather than uncertainty about animal emotions. Nevertheless, this raises the possibility of response bias, as students who completed these items may have differed from those who did not. Future studies could minimise this source of error by using simpler response formats, oral administration, or additional piloting adapted to local educational contexts.

### 4.5. Implications and Future Directions

The findings of the present survey suggest that the disconnect between students’ practical animal knowledge and formal curricula represents an important opportunity for educational innovation. The ABEK program’s success in integrating pastoral livelihoods demonstrates its feasibility [13,14]. Curricula might validate children’s experiential knowledge by creating frameworks for articulating behavioural observations and traditional ecological knowledge, while bridging practical and scientific knowledge systems through ethology and animal welfare science connected to daily experiences.

Future research should: (1) employ ethnographic observation of children’s daily interactions with animals to ground quantitative findings, (2) use longitudinal designs tracking knowledge development as children assume greater responsibilities, (3) conduct comparative analyses across urban vs. pastoral contexts or different pastoral systems, (4) examine gender differences in animal-related knowledge and practices, and (5) directly assess animal welfare in relation to children’s knowledge and beliefs.

Methodologically, cross-cultural human–animal relations research must avoid assuming Western frameworks are universal, should employ mixed methods combining breadth and depth, centre local epistemologies, examine within-culture diversity, and critically reflect on researcher positionality and power dynamics. Future studies should complement measures of emotion attribution with observational or scenario-based assessments of emotion recognition, in order to better understand how children’s beliefs about animal emotions relate to their ability to interpret animals’ behavioural cues in real-world situations.

## 5. Conclusions

Students in pastoral Karamoja demonstrate widespread recognition of animals as emotional and cognitive beings, articulated primarily through observable behavioural expressions. Patterns of emotional attribution differed more by emotion type than by species, and where species differences emerged, they reflected animals’ functional roles rather than abstract hierarchies. This knowledge appears grounded in daily practical experience rather than formal education.

These findings challenge researchers to move beyond Western, pet-centred frameworks when examining human–animal relations in pastoral contexts. Emotional attribution does not map uniformly onto standardised empathy measures, and the absence of sentimentalised language does not indicate a lack of understanding. Instead, pastoral students’ animal knowledge represents situated, functional expertise adapted to their socio-ecological realities. Future research should employ culturally grounded, methodologically pluralistic approaches to respect the diversity of ways humans and animals coexist across the world’s ecological and cultural contexts.

## Figures and Tables

**Table 1:** Students’ perceptions of the uses and social roles of different animal species. Values are presented as number of respondents (n) and column percentages (%), followed by 95% Wald confidence intervals for the estimated probabilities obtained from the statistical models.

Question	Sheep/Goats	Cattle	Chickens	Dogs	Donkeys	*p*-Value
Source of blood for consumption	92 ^a^ (30.6%) [25.4–35.8%]	51 ^b^ (16.5%) [12.8–21.1%]	60 ^b^ (20.1%) [16.0–25.1%]	N/A	43 ^b^ (14.5%) [11.0–19.1%]	<0.001
Source of meat	258 ^a^ (85.4%) [80.9–88.9%]	241 ^a^ (77.5%) [72.4–81.7%]	253 ^a^ (83.2%) [78.5–87.0%]	N/A	117 ^b^ (39.9%) [34.6–45.8%]	<0.001
Source of milk/eggs	183 ^a^ (60.4%) [54.6–65.6%]	206 ^a^ (66.5%) [60.9–71.4%]	248 ^b^ (80.5%) [75.6–84.5%]	N/A	76 ^c^ (25.9%) [21.3–31.4%]	<0.001
Skin/feathers for clothing	181 ^ab^ (59.5%) [54.1–65.1%]	173 ^ab^ (56.4%) [60.9–71.4%]	148 ^ab^ (48.8%) [43.1–54.3%]	N/A	132 ^b^ (44.4%) [39.0–50.3%]	<0.001
Skin/feathers for bedding or housing	145 ^a^ (48.0%) [42.6–53.8%]	142 ^a^ (46.1%) [40.7–51.9%]	100 ^b^ (33.0%) [27.7–38.3%]	N/A	101 ^b^ (33.9%) [28.8–39.6%]	<0.001
Marriage ceremonies	218 ^a^ (71.7%) [66.3–76.4%]	210 ^a^ (69.1%) [63.9–74.2%]	155 ^b^ (52.0%) [46.2–57.5%]	N/A	104 ^c^ (35.5%) [30.0–40.9%]	<0.001
Initiation ceremonies	151 ^a^ (52.4%) [46.5–58.0%]	145 ^a^ (50.3%) [44.8–56.3%]	137 ^a^ (47.4%) [41.9–53.3%]	N/A	100 ^b^ (34.6%) [29.5–40.4%]	<0.001
Village protection	101 ^a^ (34.4%) [28.9–39.7%]	117 ^a^ (39.0%) [33.4–44.4%]	113 ^a^ (38.7%) [33.4–44.6%]	207 ^b^ (70.4%) [64.8–75.3%]	84 ^a^ (28.9%) [23.7–34.1%]	<0.001
Not kept	133 ^a^ (49.1%) [43.0–54.8%]	123 ^a^ (44.4%) [38.8–50.5%]	149 ^a^ (55.2%) [49.4–61.2%]	117 ^a^ (43.7%) [37.6–49.5%]	60 ^b^ (21.9%) [17.5–27.3%]	<0.001

Each animal species was evaluated through a separate yes/no question; therefore, respondents could provide affirmative responses for more than one species. Different lowercase superscript letters within a row indicate significant pairwise differences among animal species (*p* < 0.05). *p*-values refer to the overall generalised linear model. N/A, not applicable.

**Table 2:** Number of students (n) and estimated marginal means (%) attributing specific emotions to different animal species, with their 95% Wald confidence intervals [95% CIs]. The Overall column reports the total number of affirmative responses and the corresponding estimated marginal percentage for each emotion across all animal species. *p*-values refer to the overall effect of animal species within each emotion.

Emotion	Overall	Sheep/Goats	Cats	Cattle	Chickens	Dogs	Donkeys	*p*-Value
Feel anger	1374 ^A^ (79.6%) [77.7–81.5%]	242 (83.4%) [78.7–87.3%]	224 (79.4%) [74.3–83.8%]	233 (78.7%) [73.7–83.0%]	229 (79.0%) [73.9–83.3%]	225 (79.5%) [74.4–83.8%]	221 (77.3%) [72.1–81.8%]	0.522
Feel disgust	1026 ^E^ (63.2%) [60.8–65.5%]	170 (62.5%) [56.6–68.1%]	166 (62.4%) [56.4–68.0%]	168 (60.6%) [54.8–66.2%]	176 (64.5%) [58.6–69.9%]	174 (64.9%) [59.0–70.4%]	172 (64.2%) [58.3–69.7%]	0.908
Feel happiness	1392 ^A^ (78.2%) [76.5–80.4%]	248 ^a^ (83.8%) [79.1–87.6%]	223 ^b^ (75.6%) [70.4–80.2%]	229 ^b^ (76.3%) [71.2–80.8%]	247 ^a^ (83.7%) [79.1–87.5%]	222 ^b^ (74.7%) [69.5–79.4%]	223 ^b^ (75.1%) [69.9–79.7%]	0.005
Feel hatred towards another animal	1189 ^C^ (71.2%) [69.0–73.4%]	200 (71.2%) [65.6–76.2%]	203 (73.8%) [68.3–78.7%]	187 (66.1%) [60.4–71.4%]	202 (71.4%) [65.8–76.3%]	200 (72.5%) [66.9–77.4%]	197 (72.2%) [66.5–77.2%]	0.442
Feel jealousy towards another animal	1131 ^D^ (69.0%) [66.8–71.3%]	181 ^b^ (64.6%) [58.9–70.0%]	183 ^ab^ (69.1%) [63.2–74.3%]	187 ^b^ (66.3%) [60.6–71.6%]	204 ^a^ (73.9%) [68.4–78.8%]	194 ^ab^ (72.4%) [66.7–77.4%]	182 ^ab^ (67.9%) [62.1–73.2%]	0.151
Feel loneliness	1141 ^D^ (68.9%) [66.6–71.1%]	193 (68.9%) [63.3–74.1%]	187 (69.3%) [63.5–74.5%]	201 (70.3%) [64.7–75.3%]	192 (69.1%) [63.4–74.2%]	195 (71.4%) [65.8–76.5%]	173 (64.3%) [58.4–69.8%]	0.591
Feel love towards another animal	1281 ^C^ (72.8%) [70.9–75.1%]	237 ^a^ (78.7%) [73.8–83.0%]	192 ^b^ (66.4%) [60.8–71.6%]	224 ^a^ (76.2%) [71.0–80.7%]	229 ^a^ (77.4%) [72.2–81.8%]	191 ^b^ (65.9%) [60.2–71.1%]	208 ^ab^ (71.7%) [66.3–76.6%]	<0.001
Feel sadness	1284 ^B^ (76.3%) [74.3–78.4%]	224 (78.9%) [73.7–83.2%]	210 (76.4%) [71.0–81.0%]	214 (74.8%) [69.5–79.5%]	207 (73.1%) [67.7–78.0%]	220 (78.9%) [73.7–83.2%]	209 (76.0%) [70.6–80.7%]	0.556
Feel satisfaction	1216 ^B^ (75.8%) [73.6–77.8%]	214 (78.4%) [73.1–82.9%]	195 (75.0%) [69.4–79.9%]	207 (75.3%) [69.8–80.0%]	202 (75.4%) [69.9–80.2%]	200 (75.5%) [69.9–80.3%]	198 (75.0%) [69.4–79.9%]	0.939
Feel fear	1239 ^B^ (73.8%) [71.8–76.0%]	221 ^a^ (78.4%) [73.2–82.8%]	201 ^ab^ (73.1%) [67.5–78.0%]	218 ^a^ (75.7%) [70.4–80.3%]	223 ^a^ (78.2%) [73.1–82.7%]	179 ^b^ (65.8%) [60.0–71.2%]	197 ^ab^ (71.4%) [65.8–76.4%]	0.006
Feel surprise	1139 ^D^ (67.4%) [65.2–69.7%]	190 (66.7%) [61.0–71.9%]	193 (70.4%) [64.8–75.5%]	185 (63.4%) [57.7–68.7%]	194 (69.0%) [63.4–74.2%]	189 (67.7%) [62.0–73.0%]	188 (67.4%) [61.7–72.6%]	0.589
Feel pain	996 ^B^ (72.9%) [70.5–75.3%]	171 (75.3%) [69.3–80.5%]	169 (74.1%) [68.0–79.4%]	161 (70.0%) [63.8–75.6%]	161 (70.6%) [64.4–76.2%]	178 (77.7%) [71.9–82.7%]	156 (69.3%) [63.0–75.0%]	0.248

Each animal species was evaluated through a separate yes/no question; therefore, respondents could provide affirmative responses for more than one species. Different uppercase superscript letters in the Overall column indicate significant differences among emotion types (*p* < 0.05). Different lowercase letters within a row indicate significant pairwise differences among animal species (*p* < 0.05); superscripts are shown only when at least one pairwise comparison was significant.

**Table 3:** Number of affirmative responses (n) and estimated marginal means (%) for the attribution of cognitive and functional abilities to different animal species, with their 95% Wald confidence intervals [95% CIs]. The Overall column reports the total number of affirmative responses and the corresponding estimated marginal mean across all animal species. *p*-values refer to the overall effect of animal species within each ability.

Ability	Overall	Sheep/Goats	Cats	Cattle	Chickens	Dogs	Donkeys	*p*-Value
Defend the herd	977 (62.9%) [60.5–65.3%]	163 (62.9%) [56.9–68.6%]	174 (67.4%) [61.5–72.9%]	149 (56.2%) [50.2–62.1%]	165 (64.0%) [57.9–69.6%]	165 (63.7%) [57.7–69.3%]	161 (63.1%) [57.0–68.8%]	0.186
Defend themselves	1101 (69.5%) [67.3–71.8%]	188 (70.9%) [65.2–76.1%]	187 (71.6%) [65.9–76.8%]	185 (68.3%) [62.5–73.5%]	172 (65.9%) [59.9–71.4%]	194 (73.8%) [68.1–78.7%]	175 (66.5%) [60.6–72.0%]	0.312
Feel love for their farmer	1163 (73.0%) [70.8–75.2%]	195 (73.6%) [67.9–78.5%]	189 (71.9%) [66.1–77.0%]	217 (79.8%) [74.6–84.1%]	187 (70.6%) [64.8–75.7%]	193 (73.1%) [67.4–78.1%]	182 (68.7%) [62.8–74.0%]	0.083
Find a source of water	1310 (82.3%) [81.0–84.8%]	237 ^a^ (89.4%) [85.1–92.6%]	192 ^b^ (73.3%) [67.6–78.3%]	240 ^a^ (87.3%) [82.8–90.7%]	205 ^b^ (77.7%) [72.2–82.3%]	217 ^a^ (82.5%) [77.4–86.6%]	219 ^a^ (83.6%) [78.6–87.6%]	<0.001
Help each other	999 (62.0%) [59.7–64.4%]	169 (63.1%) [57.1–68.6%]	149 (56.0%) [50.0–61.9%]	168 (60.6%) [54.8–66.2%]	171 (64.0%) [58.1–69.6%]	172 (64.4%) [58.5–69.9%]	170 (63.9%) [58.0–69.5%]	0.315
Mothers recognise their offspring	1270 (78.8%) [76.8–80.8%]	214 (79.6%) [74.3–84.0%]	213 (80.4%) [75.2–84.7%]	213 (76.3%) [71.0–81.0%]	205 (77.1%) [71.6–81.7%]	217 (81.0%) [75.8–85.2%]	208 (78.8%) [73.4–83.3%]	0.743
Recognise animals from the same herd	1033 (67.0%) [64.6–69.3%]	177 (67.8%) [61.9–73.2%]	164 (64.6%) [58.5–70.2%]	175 (66.5%) [60.6–72.0%]	173 (67.6%) [61.6–73.0%]	172 (67.7%) [61.7–73.2%]	172 (67.7%) [61.7–73.2%]	0.967
Recognise danger	996 (62.5%) [60.4–65.3%]	149 ^b^ (55.6%) [49.6–61.4%]	188 ^a^ (71.8%) [66.0–76.9%]	139 ^b^ (51.9%) [45.9–57.8%]	165 ^b^ (61.8%) [55.8–67.4%]	196 ^a^ (73.4%) [67.8–78.4%]	159 ^b^ (60.7%) [54.6–66.4%]	<0.001
Recognise their farmer	1084 (69.1%) [66.8–71.4%]	186 (70.7%) [64.9–75.9%]	173 (66.8%) [60.8–72.3%]	184 (67.9%) [62.1–73.2%]	188 (72.3%) [66.6–77.4%]	177 (69.1%) [63.2–74.5%]	176 (67.7%) [61.8–73.1%]	0.755
Return alone to the village	1128 (67.5%) [65.8–70.4%]	189 ^b^ (67.7%) [62.0–73.0%]	194 ^b^ (70.3%) [64.6–75.4%]	167 ^c^ (58.4%) [52.6–64.0%]	199 ^b^ (71.8%) [66.3–76.8%]	225 ^a^ (80.6%) [75.6–84.9%]	154 ^c^ (56.4%) [50.5–62.2%]	<0.001
Take care of other animals	1057 (66.3%) [63.9–68.6%]	179 (67.0%) [61.2–72.4%]	169 (64.0%) [58.0–69.6%]	178 (65.7%) [59.8–71.1%]	182 (68.9%) [63.1–74.2%]	178 (67.2%) [61.3–72.6%]	171 (64.8%) [58.8–70.3%]	0.861
Take care of themselves	1222 (75.8%) [73.7–77.9%]	198 (73.3%) [67.7–78.3%]	201 (75.0%) [69.5–79.8%]	208 (75.9%) [70.5–80.6%]	201 (75.8%) [70.3–80.6%]	213 (78.6%) [73.3–83.1%]	201 (76.1%) [70.6–80.9%]	0.824

Each animal species was evaluated through a separate yes/no question; therefore, respondents could provide affirmative responses for more than one species. Different lowercase letters within a row indicate significant pairwise differences among animal species (*p* < 0.05); letters are shown only when at least one pairwise comparison was significant. *p*-values refer to the overall effect of animal species within each ability.

**Table 4:** Pairwise associations among students’ reported behaviours when encountering an unfamiliar dog. Behaviour pairs are ordered by increasing Benjamini–Hochberg false discovery rate (FDR)-adjusted *p*-values and are presented as odds ratios (OR) with 95% confidence intervals (95% CI).

Behaviour Pair	OR (95% CI)	*p*-Value	FDR-Adjusted *p*
Let dog sniff hand ↔ Chase dog away	3.31 (2.03–5.41)	<0.001	**0.003**
Ask owner ↔ Would not approach	2.70 (1.52–4.77)	0.001	**0.003**
Let dog sniff hand ↔ Ask owner	2.48 (1.43–4.31)	0.001	**0.003**
Let dog sniff hand ↔ Would not approach	2.41 (1.50–3.88)	<0.001	**0.003**
Call dog gently ↔ Would not approach	2.19 (1.35–3.57)	0.001	**0.003**
Chase dog away ↔ Would not approach	1.99 (1.24–3.20)	0.004	**0.010**
Touch head ↔ Call dog gently	1.90 (1.17–3.10)	0.010	**0.021**
Call dog gently ↔ Ask owner	1.84 (1.08–3.13)	0.024	**0.045**
Touch head ↔ Let dog sniff hand	1.59 (0.98–2.58)	0.061	0.083
Call dog gently ↔ Chase dog away	1.61 (0.99–2.61)	0.053	0.080
Ask owner ↔ Chase dog away	1.55 (0.90–2.67)	0.117	0.146
Touch head ↔ Chase dog away	1.14 (0.70–1.85)	0.610	0.656
Touch head ↔ Would not approach	0.88 (0.55–1.43)	0.612	0.656
Touch head ↔ Ask owner	0.92 (0.53–1.59)	0.762	0.762

Odds ratios (OR) > 1 indicate positive co-occurrence between the two reported behaviours. Pairwise associations were evaluated using Pearson’s chi-square test. *p*-values were adjusted for multiple comparisons using the Benjamini–Hochberg false discovery rate (FDR) procedure. Bold values indicate associations that remained statistically significant after FDR correction.

**Table 5:** Pairwise associations among students’ reported behaviours when faced with a growling dog. Behaviour pairs are ordered by increasing Benjamini–Hochberg false discovery rate (FDR)-adjusted *p*-values and are presented as odds ratios (ORs) with 95% confidence intervals (95% CIs).

Behaviour Pair	OR (95% CI)	*p*	FDR-Adjusted *p*
Run away ↔ Look for help	3.74 (2.14–6.53)	<0.001	**0.003**
Run away ↔ Try to fight the dog off	3.29 (1.76–6.15)	<0.001	**0.003**
Look for help ↔ Try to fight the dog off	2.22 (1.14–4.32)	0.017	0.057
Try to calm the dog by talking gently ↔ Look for help	1.80 (1.04–3.13)	0.035	0.088
Run away ↔ Stay still without looking at the dog in the eyes	0.62 (0.38–1.02)	0.058	0.089
Stay still without looking at the dog in the eyes ↔ Try to calm the dog by talking gently	1.57 (0.98–2.52)	0.060	0.089
Try to calm the dog by talking gently ↔ Try to fight the dog off	1.63 (0.97–2.73)	0.062	0.089
Stay still without looking at the dog in the eyes ↔ Try to fight the dog off	1.31 (0.78–2.19)	0.312	0.390
Stay still without looking at the dog in the eyes ↔ Look for help	1.27 (0.74–2.16)	0.387	0.430
Run away ↔ Try to calm the dog by talking gently	0.99 (0.61–1.61)	0.966	0.966

Odds ratios (ORs) > 1 indicate positive co-occurrence between the two reported behaviours. Pairwise associations were evaluated using Pearson’s chi-square test. *p*-values were adjusted for multiple comparisons using the Benjamini–Hochberg false discovery rate (FDR) procedure. Bold values indicate associations that remained statistically significant after FDR correction.

## Data Availability

The data are not publicly available due to privacy and confidentiality restrictions related to the ALL IN ONE Project, an international cooperation project involving multiple partners and funded by the Italian Agency for Development Cooperation (AICS). The data supporting the findings of this study are available from the corresponding author upon reasonable request and with the permission of the project partners, subject to the project’s confidentiality requirements.

## Supplementary Materials

The following supporting information can be downloaded at https://www.mdpi.com/article/10.3390/ani16182812/s1, Document S1: Student Questionnaire. Full questionnaire administered to participating students, including all sections and items as included in the final version of the instrument; Document S2: Data Entry, Cleaning, and Coding Procedures. Detailed description of the data entry, dataset cleaning, and response coding procedures; Table S1: Coding Categories for Open-Ended Responses. Coding scheme used to classify and allocate responses to open-ended questions, with a description of each category and the corresponding answer included; Tables S2–S6: Detailed Statistical Results. Complete analytical results not reported in the main text to improve readability.
