# Supplementary material for: Students’ Conceptions of Animal Emotions and Cognitive Abilities in Karamoja (Uganda): Implications for Animal Welfare Education and One Health
Source: Animals (Basel). 2026 Sep 8;16(18):2812. doi: 10.3390/ani16182812 (PMC13603455; doi:10.3390/ani16182812)
Supplement: Supplementary file 1 [file animals-16-02812-s001.zip › Document S1.pdf]

Supplementary Material: Document S1

HUMAN ANIMAL EMOTIONS QUESTIONNAIRE

Name interviewed ____ Age ____ Sex _____________

What is your family language? ___________________At what age did you start going to school?_________

How many siblings do you have?_________________________

Who do you live with?   parent(s)   other family members

Who do you feel closer to among your family members?________________________

How do you help your family?

I take the animals to graze
I go get the water to the borehole
I cook
I sell things on the street
I collect firewood
I work in the fields
I clean the house
I do not help my family in any way
other……………………………………………………..

Is your father a farmer? Yes   No   Which animals does your family own?_______________________________

1) How much are animals important for your family?

Not important at all   Of little importance   Moderately important   Important   Very important

Why? ____

2) What are the animals in your family used for?

	Drink blood	Eat meat	Eat milk/ eggs	Skin/feathers for dressing	Skin/feathers for bed/house	Marriage	Initiations	Village protection	Don't have
Cattle									
Sheep/Goats									
Chickens									
Donkey									
Dogs									

3) Do you think animals can communicate between them?   Yes   No

4) If yes, how do they communicate?

____

5) Can you describe what an animal does when…

angry ____

scared ____

happy____

sad____

surprised____

disguested____--

6) Do you love animals? Yes No 7) Do you think animals can love you? Yes No

8) If yes, which animals do you think can love you?

____

9) Which is your favourite animal?____

10) Do you play with animals? Yes No If yes, how do you play with them?

____

11) Do you play with dogs? Yes No If yes, how do you play with them?

____

12) Are you afraid of dogs? Yes No If yes, why?

____

13) Do you think animals can think? Yes No If yes, which is the most intelligent (1st, 2nd, etc.)?
Cattle Sheep/Goats Chickens Donkey Dogs

14) Do you study animals at school? Yes No If yes, what do you study? ______________________

____

____

____

16) Can an animal… (write YES or NO for each of the following animals)

	Cattle	Sheep/Goats	Chickens	Donkey	Dogs	Cats
feel happy						
feel love for another animal						
feel angry						
feel scared						
feel surprised by something unexpected						
feel disgusted by something he/she does not						
feel lonely						
feel sad						

feel hateful towards another animal						
feel jealous of another [illegible]						
feel satisfied						
which animals can feel [illegible]						

17) Can an animal… (write YES or NO for each of the following animals)

	Cattle	Sheep/ Goats	Chickens	Donkeys	Dogs	Cats
return alone to the village						
recognize the danger						
mothers recognize their kids						
recognize the animals of the same herd						
recognize their farmers						
feel love for the farmer						
help each others						
defend themselves						
defend the herd						
take care of themselves						
take care of other animals						
find a source of water						

18) How would you you approach an unfamiliar dog?

- ☐ I touch the head of the dog with my hand
- ☐ I call the dog gently and I look it in the eyes
- ☐ I leave the dog sniffing my hand before to touch it
- ☐ I ask to the owner if the dog is good and if I can touch it
- ☐ I chase the dog away by shouting, throwing stones, etc.
- ☐ I would not approach an unfamiliar dog

19) What would you if you are in front of a dog that growls?

- ☐ I run away
- ☐ I stay still without looking at the dog in the eyes
- ☐ I try to calm the dog gently talking
- ☐ I look for help
- ☐ I try to fight the dog off
