# Supplementary material for: Students’ Conceptions of Animal Emotions and Cognitive Abilities in Karamoja (Uganda): Implications for Animal Welfare Education and One Health
Source: Animals (Basel). 2026 Sep 8;16(18):2812. doi: 10.3390/ani16182812 (PMC13603455; doi:10.3390/ani16182812)
Supplement: Supplementary file 1 [file animals-16-02812-s001.zip › Document S2.pdf]

Supplementary Material: Document S2

In depth description of the workflow, from data entry to data curation

Data Entry

All questionnaires were transcribed into a structured Microsoft Excel database following field collection. Open-ended responses were transcribed verbatim to preserve the children's original wording and enable subsequent qualitative analysis.

Closed-ended yes/no questions were coded using a binary format (1 = "yes", 0 = "no"). For matrix-type questions, each species–attribute combination was entered as a separate variable, with responses coded as 1 (selected) or 0 (not selected). Likert-type responses were converted into numerical scores (not important at all = 1; of little importance = 2; moderately important = 3; important = 4; very important = 5).

Missing responses were left as blank cells. Responses that were present but impossible to interpret because they were incoherent, unintelligible, or unrelated to the question were coded as NV (non-valid), allowing them to be distinguished from both valid responses and true missing values.

For all structured variables (binary, matrix-type, and Likert-type items), only predefined admissible values were permitted, thereby preventing the accidental introduction of inconsistent entries.

Data Cleaning

Following data entry, the entire database underwent a structured data-cleaning procedure to verify internal consistency and prepare the dataset for statistical analyses.

During this process, responses originally entered as free text were reviewed to identify entries that were unintelligible, contradictory, or unrelated to the question. These responses were reclassified as NV (non-valid) where appropriate, ensuring a clear distinction between meaningful qualitative responses, invalid responses, and missing data.

Qualitative Data Curation

Open-ended responses were reviewed line by line and, for each question, inductively grouped into thematic categories based on shared semantic content. The categories were developed by examining recurring concepts expressed by the participants rather than by applying predefined ethological classifications.

The thematic categories were intended to summarise the children's responses rather than to classify behaviours according to formal ethological definitions. Consequently, some categories reflect children's perceptions or interpretations of animal behaviour, which do not necessarily correspond to scientifically recognised behavioural indicators.

Each category encompassed multiple variants of children's wording. For example, responses referring to running, jumping, or energetic play were grouped under Movement/Play, whereas expressions such as crying, looking sad, or being unhappy were grouped under Crying/Vocal expression/Sadness. Additional examples of the thematic coding are provided in the Supplementary Tables.

A residual category ("Other") was created for interpretable responses that did not fit conceptually within any of the predefined thematic groups. This category also included responses describing internal states, contextual situations, or meaningful statements that did not represent observable animal behaviours. Inclusion in this category followed two criteria: (1) the response appeared fewer than five times across the entire dataset, indicating insufficient recurrence to justify the creation of a standalone category; and (2) the content could not be semantically assigned to any of the existing themes. This approach allowed preservation of low-frequency but interpretable responses while avoiding the creation of overly fragmented categories with limited analytical value.
