# Supplementary material for: Students’ Conceptions of Animal Emotions and Cognitive Abilities in Karamoja (Uganda): Implications for Animal Welfare Education and One Health
Source: Animals (Basel). 2026 Sep 8;16(18):2812. doi: 10.3390/ani16182812 (PMC13603455; doi:10.3390/ani16182812)
Supplement: Supplementary file 1 [file animals-16-02812-s001.zip › Table S1.pdf]

Supplementary Material: Table S1

The table displays the semantic categorisation of the answer for each open-ended question.

Category	Words
**Q: Family language**	
Acholi	
Ateso	
Bagesu	
English	
Iugisu	
Karamojong	
Local Language	
Other	Sapei, Pokot, Pour, Magada, Basedeyi, Latin, French, Kumam, Japadola, Kasimeri, Kupsabiny, Langi, Luganda, Swahili
No sense / Invalid	
**Q: Which animals does your family own?**	
Cat	
Cattle	
Small Ruminants	
Dogs	
Other Livestock	Donkey, Pigs, Rabbit, Chicken, Ducks, Birds
More than one species	the respondent mentioned more than one species
**Q: Why? (referred to Q "How much are animals important for your family?")**	
Food	
Food and Income	
Income	
Protection	
Other	marriage, materials, cultural reason, little/not important, for help, don't have animals, digging, crops
No sense / Invalid	NV, animal names
**Q: How do they (animals) communicate?**	
By sounds / Vocalisations	making sounds, barking, shouting, crying, whistling, talking
Body language / Movement /Physical touch	seeing one another, playing, touching their noses
By social behavior / Cooperation	moving together, calling one another

Other	biting, fighting, general "communicate", communicate when angry, to love, when they want food, eating
No sense/Invalid	NV, yes, no

Q: What does an animal do when angry?	
Aggression / attack / destruction	fighting, biting, destroys things, killing, chase someone
Vocalization / sound	barking, making sounds,
Looking for food/ eating	look for food, eating
Other	behave strange, dies, annoyed, play, happy, scared, moving, running, hiding, lonely, SR
No sense/Invalid	NV, yes, no, animal types

Q: What does an animal do when scared?	
Defensive aggression	fighting, killing, biting, angry, growling
Escape / avoidance	running, hiding, alone
Vocalization / sound	barking, shouting, making sounds
Sad / scared	sad, scared, fear, crying
Other	hungry, happy, smile, sickness, not eating food, when you hit him, comes to you, stand, sleep, lie, because of seeing something, change face expression
No sense/Invalid	NV, yes, no, animal types

Q: What does an animal do when happy?	
Movement / Play	jump, run, play, dance
Feeding / Satisfaction	eating food, when it is satisfied
Smile / Positive expression	smile, happy
Other	love each other, come closer, angry, biting, making sounds, sit, sleep, SR
No sense / Invalid	yes, no, NV, animal types

Q: What does an animal do when sad?

Aggressive response/ Negative expression	fighting, biting, looks ugly, barking, can kill, can eat other, will beat
Crying / Vocal expression / Sadness	cry, making sounds, not happy, sad
Withdrawal / Isolation / Inactivity	stays alone, sleep, sit, run away, run, not playing, not eating
Other	looking for food, hungry, cannot smile/communicate, smile, happy, not feel well, feels sick, when they see the enemy, scared, hide
No sense / Invalid	NV, no, yes, animal types

**Q: What does an animal do when surprised?**	
Positive surprise / Play / Movement	smile, happy, play, run, jump
Negative surprise / Fear / Escape	when the enemy appears, running, scared, hide
Defensive / Aggressive reaction	bite, fight
Vocalization / Sound	make noise, cry, barking
Other	give each other something, when they produce, when something happens, looks at the thing, sickness, eats, sitting, sleeping, surprised
No sense / Invalid	yes, no, NV, animal types

**Q: What does an animal do when disgusted?**	
Anger / Irritation	fight, angry, bite, annoyed
Avoidance / Withdrawal	run away, run, sits alone, separates from other, don't tal to each other
Sadness / Unhappiness	cry, sad, unhappy
Other	same as people, stop moving, sleeps, vomits, sick, defecate, smile, happy, when they see each other, not eat, the dog is full, shouting, disgusted
No sense/Invalid	yes, no, animal types, NV

**Q: If yes, which animals can love you?**

Different domestic animals	livestock+dog/cat
Ruminants	cattle, small ruminants
Other	donkey, pigs, poultry
No sense/Invalid	NV, no

Q: Which is your favourite animal?	
Ruminants	cattle, small ruminants
Dog	
Cat	
Other	donkey, rabbit, pigs, wild animal, fish, poultry, none, dog+cat
No sense/Invalid	NV

Q: If yes, how do you play with them?	
Generic/Positive statement	play with them, make me happy
Feeding / Food-mediated play	giving food, by grazing, playing with water
Movement / Active play	running, jumping, chasing, throwing objects
Physical interaction	touching, scratching, sitting on them,
Other	shaking head/tail, not playing, by not being near them, by bathing, harmful reaction, traning, calling them
No sense/Invalid	NV, dog

Q: If yes, how do you play with dogs?	
Movement / Active play	running, jumping, chasing, throwing objects, dancing
Physical contact	touching, scratching, sleeping with them, sitting on them,
Feeding-mediated play	giving food, by rearing, feeding
Harmful / Fear / Avoidance	can bite, will fight, they scare, it is bad to play with

Vocal / Communication play	by calling their names, by clapping hand for them, by whistling
Other	training, generic play
No sense / Invalid	NV, no

**Q: If yes, why? (referred to Q "are you afraid of dogs?")**	
Aggression	can bite, dangerous, can chase you, can kill, when they bark
Generic fear	I fear them, because they can make you cry, I don't like looking them in their eyes, they can hate
No fear / Positive aspects	I know that they cannot kill me, they love me, they are my friend, because I like giving them food, because it guides me, because they are good for society
Other	fear of animal nature
No sense / Invalid	NV

**Q If yes, what do you study?**	
Behaviour / Emotions / Communications	study their sounds, emotions, how they feel, behaviour,
Body parts / Biology / Physiology	how animal grows, digestive system, body parts, their young ones, animals as living things, naming parts
Taxonomy	types of animals, categorization, naming animals
Care, disease, husbandry	how to keep animals, how to prevent diseases, how to take care, how/ what they produce
General study	general study of animals
No sense / Invalid	NV
