# Supplementary material for: Students’ Conceptions of Animal Emotions and Cognitive Abilities in Karamoja (Uganda): Implications for Animal Welfare Education and One Health
Source: Animals (Basel). 2026 Sep 8;16(18):2812. doi: 10.3390/ani16182812 (PMC13603455; doi:10.3390/ani16182812)
Supplement: Supplementary file 1 [file animals-16-02812-s001.zip › Tables S2-S6.pdf]

Supplementary Material: Tables S2-S6

Detailed results of the statistical analysis

Table S2: Distribution of responses to selected sociodemographic questions among the participating students. Categories within each question are presented in descending order of frequency (%).

Question	Category	Count	Column N (%)
**0.4 Family language**	Karamojong	114	35.1
	Ateso	100	30.8
	English	64	19.7
	Other	16	4.9
	Acholi	11	3.4
	Iugisu	8	2.5
	Local language	7	2.2
	Non-Valid	5	1.5
	Bagesu	0	0.0
**0.7 Who do you live with?**	Parent(s)	176	70.1
	Other family members	62	24.7
	Both	8	3.2
	Non-Valid	5	2.0
**0.8 Who are you closer to among your family members?**	Mother	156	50.5
	Father	75	24.3
	Non-Valid	24	7.8
	Other family members	18	5.8
	Parents	17	5.5
	Brother	9	2.9
	Sister	7	2.3
	No one	2	0.6
	Mother, father and children	1	0.3
**0.10 Is your father a farmer?**	Yes	144	50.7
	No	140	49.3
**0.11 Which animals does your family own?**	Cattle	110	41.8
	Small ruminants	76	28.9
	Other livestock	29	11.0
	More than one species	23	8.7
	Dogs	13	4.9
	Non-Valid	9	3.4
	Cat	3	1.1
**1.0 How important are animals for your family? (score from 1 to 5)**	5	190	70.1
	Non-Valid	23	8.5
	1	19	7.0
	2	17	6.3
	4	15	5.5
	3	7	2.6
**1.1 Why?**	Food	125	51.2
	Income	56	23.0
	Other	28	11.5
	No sense/invalid	27	11.1
	Food/income	4	1.6
	Protection	4	1.6

Table S3: Distribution of students' responses to questions investigating attitudes toward and perceptions of animal behaviour and communication. Categories within each question are presented in descending order of frequency (%).

Question	Category	Count	Column N (%)
**3.0 Do you think animals can communicate with each other?**	Yes	218	78.1
	No	61	21.9
**4.0 If yes, how do they communicate?**	By sounds/vocalizations	150	62.2
	Non-Valid	43	17.8
	Body language/movement/physical touch	29	12.0
	Other	13	5.4
	By social behaviour/cooperation	6	2.5
**5.0 What does an animal do when angry?**	Aggression/attack/destruction	84	27.7
	Non-Valid	62	20.5
	Vocalization/sound	42	13.9
	Sadness/crying	34	11.2
	Looking for food/Feeding behaviour	33	10.9
	Other	30	9.9
	Sickness/weakness/tiredness	18	5.9
**5.1 ...when scared?**	Escape/avoidance	136	48.9
	Non-Valid	63	22.7
	Other	34	12.2
	Vocalization/sound	19	6.8
	Sad/scared	15	5.4
	Defensive aggression	11	4.0
**5.2 ...when happy?**	Movement/play	143	47.2
	Non-Valid	43	14.2
	Feeding/satisfaction	37	12.2
	Smile/positive expression	33	10.9
	Self Referred*	29	9.6
	Other	18	5.9
**5.3 ...when sad?**	Aggressive/negative behavioural response	76	25.7
	Withdrawal/isolation/inactivity	73	24.7
	Crying/vocal expression/sadness	49	16.6
	Non-Valid	48	16.2
	SR	27	9.1
	Other	23	7.8
**5.4 ...when surprised?**	Positive surprise/play/movement	92	35.9
	No sense/invalid	57	22.3
	Negative surprise/fear/escape	36	14.1
	Other	34	13.3
	Self Referred*	18	7.0
	Vocalization/sound	11	4.3
	Defensive/aggressive reaction	8	3.1
**5.5 ...when disgusted?**	Non-Valid	60	27.0
	Irritation/negative reaction	42	18.9
	Other	42	18.9
	Sadness/unhappiness	36	16.2
	Avoidance/withdrawal	25	11.3
	Self Referred*	17	7.7

* *Self Refered indicates when the respondents described the emotion on themselves and not on animals.*

Table S4: Distribution of students' responses to questions exploring their relationships with animals, perceived animals' capacity to love and think, and interactions with dogs. Categories within each question are presented in descending order of frequency (%).

Question	Category	Count	Column N (%)
**6. Do you love animals?**	Yes	291	97.7
	No	7	2.3
**7. Do you think animals can love you?**	Yes	215	82.1
	No	47	17.9
**8. If yes, which animals can love you?**	Dog	197	65.0
	Ruminants	42	13.9
	Cat	26	8.6
	Different domestic animals	11	3.6
	Dog and cat	9	3.0
	Non-Valid	9	3.0
	Other	9	3.0
**9. Which is your favourite animal?**	Ruminants	120	47.2
	Dog	80	31.5
	Other	25	9.8
	Cat	19	7.5
	Non-Valid	10	3.9
**10. Do you play with animals?**	Yes	232	77.6
	No	67	22.4
**10.1 If yes, how do you play with them?**	Movement/active play	125	55.8
	Feeding/food-mediated play	34	15.2
	Non-Valid	21	9.4
	Other	17	7.6
	Generic/positive statement	16	7.1
	Physical contact	11	4.9
**11. Do you play with dogs?**	Yes	191	64.3
	No	106	35.7
**11.1 If yes, how do you play with dogs?**	Movement/active play	119	58.6
	Non-Valid	20	9.9
	Feeding-mediated play	17	8.4
	Physical contact	15	7.4
	Harmful/fear/avoidance	13	6.4
	Other	12	5.9
	Vocal/communication play	7	3.4
**12. Are you afraid of dogs?**	Yes	183	65.8
	No	95	34.2
**12.1 If yes, why?**	Fear of aggression/biting	120	60.6
	Non-Valid	35	17.7
	No fear/positive aspects	34	17.2
	Generic fear	7	3.5
	Other	2	1.0
**13. Do you think animals can think?**	Yes	210	81.7
	No	47	18.3

Table S5: Students' reported behaviours in response to the question: "*How would you approach an unfamiliar dog?*" (N = 288).

**Questionnaire item**	Yes, n (%)
I touch the head of the dog with my hand.	184 (63.9%)
I call the dog gently and look it in the eyes.	171 (59.2%)
I let the dog sniff my hand before touching it.	143 (49.7%)
I ask the owner whether the dog is friendly and whether I can touch it.	213 (74.0%)
I chase the dog away by shouting, throwing stones, or similar behaviours.	122 (42.4%)
I would not approach an unfamiliar dog.	130 (45.1%)

Table S6: Students' reported behaviours in response to the question: *"What would you do if faced with a growling dog?"* (N = 285).

Questionnaire item	Yes, n (%)
**I run away.**	185 (64.9%)
**I stay still without looking the dog in the eyes.**	155 (54.4%)
**I try to calm the dog by talking gently.**	134 (47.0%)
**I look for help.**	213 (74.7%)
**I try to fight the dog off.**	83 (29.1%)
